# Outlines of a Course of Lectures on Medical Jurisprudence

**Published:** 1837-04

**Authors:** 


Art. XVI.—*Outlines of a Course of Lectures on Medical Jurisprudence.* By T. S. Traill, m.d., Regius Professor of Medical Jurisprudence in the University of Edinburgh.—*Edinburgh.* 12mo. p. 94.

This small work is reprinted from Dr. Traill's Dissertation on Medical Jurisprudence, in the seventh edition of the Encyclopædia Britannica, for the use of his pupils in the University. It is a masterly view of the subject, containing in a small compass much information, well considered, judiciously selected, and concisely expressed. To the pupils of Dr. Traill's class it is peculiarly valuable, and those who have completed their education will find it a useful help in keeping up their knowledge of medical jurisprudence, when, from want of time and practical opportunities, what they once knew is fast slipping away from them. To such persons, a short and comprehensive sketch of the chief subjects will bring the details again to their recollection. The medical coroner will frequently find such a guide useful in suggesting to him points that should be particularly attended to in the investigation he may be pursuing, at a time when reference to larger works may be difficult or impossible.

